# Heart function assessment during aging in apolipoprotein E knock-out mice

**DOI:** 10.15190/d.2021.15

**Published:** 2021-09-28

**Authors:** Elisa A. Liehn, Ana-Mihaela Lupan, Rodica Diaconu, Mihai Ioana, Ioana Streata, Catalin Manole, Alexandrina Burlacu

**Affiliations:** ^1^Human Genetic Laboratory, University of Medicine and Pharmacy of Craiova, Craiova, Romania; ^2^Department of Cardiology, Angiology and Intensive Care, Medical Faculty, University Hospital Aachen, Aachen, Germany; ^3^Victor Babes National Institute of Pathology, Bucharest, Romania; ^4^Nicolae Simionescu Institute of Cellular Biology and Pathology, Bucharest, Romania

**Keywords:** apoE, cardiovascular models, cardiomyopathy.

## Abstract

BACKGROUND: Apolipoprotein (apo) E isoforms have strong correlations with metabolic and cardiovascular diseases. However, it is not clear if apoE has a role in development of non-ischemic cardiomyopathy. Our study aims to analyze the involvement of apoE in non-ischemic cardiomyopathy. METHODS AND RESULTS: Serial echo-cardiographic measurements were performed in old wildtype and apoE deficient (apoE-/-) mice. Morphological and functional cardiac parameters were in normal range in both groups at the age of 12 month. At the age of 18 months, both groups had shown ventricular dilation and increased heart rates. However, the apoE-/- mice presented signs of diastolic dysfunction by hypertrophic changes in left ventricle, due probably to arterial hypertension. The right ventricle was not affected by age or genotype. CONCLUSION: Even in the absence of high fat diet, apoE deficiency in mice induces mild changes in the cardiac function of the left ventricle during aging, by developing diastolic dysfunction, which leads to heart failure with preserved ejection fraction. However, further studies are necessary to conclude over the role of apoE in cardiac physiology and its involvement in development of heart failure.

## INTRODUCTION

Apolipoprotein E (apoE) is a ubiquitous protein, essential for the lipid metabolism in the whole body^1,2^. The role of apoE in lipid metabolism, ensuring the fulfillment of cholesterol homeostasis and adequate clearance to circulating lipids^3,4^ is the best known. Numerous studies have shown new other functions of this protein, independent of lipid metabolism, thus highlighting the polymorphism and multifunctionality of apoE^5^. Therefore, the deep understanding of all functions of this lipoprotein is for a great interest for the current research community, especially in the field of cardiovascular and metabolic diseases.

There are three different *apoE* alleles in humans: ε2, ε3, ε4. ε3 allele is the most common with a prevalence of 62% in the population, being considered the wild-type isoform^6^. Current clinical knowledge of apoE is limited to statistical correlations of certain alleles and/or genotypes with various chronic conditions^6-10^. The progression of cardiovascular diseases such as atherosclerosis is more accelerated in carriers of certain apoE alleles^5^.

Recently, a strong correlation was observed between the presence of apoE isoforms and severe Covid-19 infections^11-15^, due to exaggerated lung inflammation in these patients. Increased susceptibility to inflammation seems also responsible for development of dilatative cardiomyopathy^16,17^ in patients carrying *e4* allele. However, a direct link between the apoE defects and development of cardiomyopathy is still under debate.

The current study aims to investigate the effect of apoE deficiency on the heart function.

## MATERIAL AND METHODS

### Animal model

All animal experiments were performed after approval (No. 359 from 19.06.2017), according to the European legislation and according to the FELASA guideline for care and use of experimental animals. Twelve-month old C57BL/6 mice (Mus Musculus, wild-type) and apoE knockout mice with matched background (apoE^-/-^) were fed normal diet (3.3% crude fat, cholesterol-free). Echocardio-graphy was performed at the beginning of experiments and 6 months later (at the age of 18 months). Mice were kept during all period in standardized conditions (21° ± 2 °C, 60% ± 5% humidity, and a 12-hour light/dark cycle, free access to food and water ad libitum).

### Study design

Nineteen mice, females and males, were included in the study as presented in the Table 1.

**Table 1:** Mice included in the study

Genotype	Sex	12 months	18 months	Died during the observation period
WILD-TYPE	female	n=4	n=3	1
	male	n=3	n=1	2
ApoE-/-	female	n=7	n=6	1
	male	n=5	n=5	0

### Echocardiography

Mice were sedated with 1.5% isoflurane, placed on a warm, ECG-monitoring table. Echocardiographic measurements were performed using a small-animal ultrasound imager (Vevo 2100, FUJIFILM Visualsonics, Toronto, Canada). Measurements in short and long parasternal axis were taken in B-Mode (2D-realtime) and M-Mode using a 40 MHz transducer, as described before^18,19^. The left ventricular (LV) ejection fraction (EF) was assessed and analyzed in the long and short axes obtained at the level of papillary muscles. The systolic and diastolic LV volumes, LV wall thickness, LV mass, heart rate (HR), as well as the valve gradients and velocity were also recorded and calculated.

### Statistical analysis

Data represent mean ± SEM. Statistical analysis was performed with Prism 6.1 software (GraphPad). Means of more than two groups were compared with 1-way ANOVA followed by Tukey’s post-hoc-test, as indicated. p-values of <0.05 were considered significant.

## RESULTS

Nineteen mice, females and males, were included in the study (Table 1). Four mice died during observation period. No correlation was made between the survival rate and the mouse’s genotype. There were no differences between the males and female’s echocardiographic parameters within each group, therefore, the results included all mice, independent of gender.

**Figure 1:**
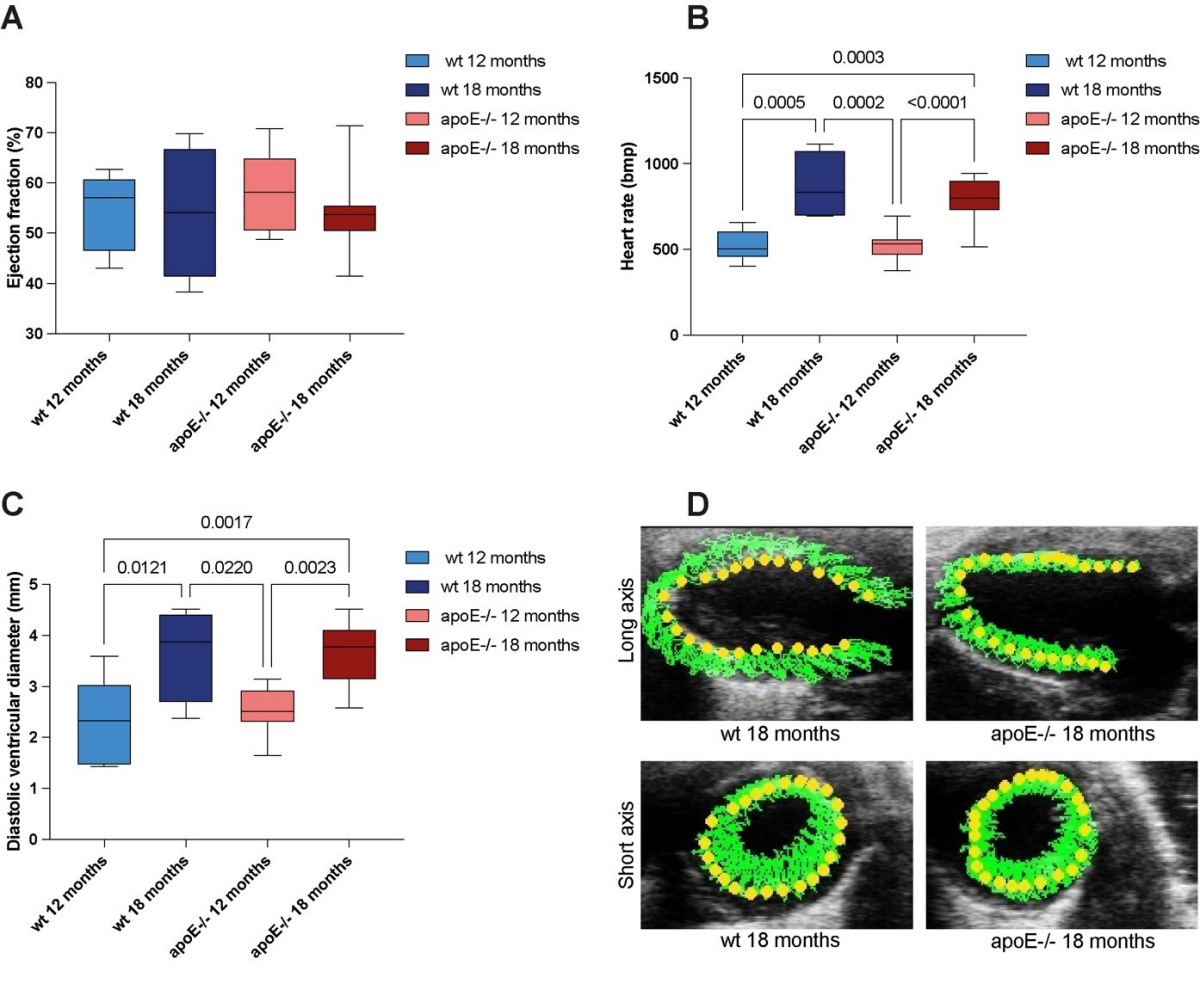
Morphological and functional assessment of the heart. (**A**) Ejection fraction, (**B**) Heart rate and and (**C**) Diastolic ventricular diameter of the mice measured at 12 and 18-months old. (**D**) Representative strain ultrasound images in long axis (upper panel) and short axis (lower panel). Significant *p *values are depicted in each figure.

During aging, both the wildtype and apoE^-/-^ mice have lost weight, but this was not statistically different. The EF also remained preserved over the time in both groups (Figure 1A). However, the mice present a significant increase in the heart rate during aging (Figure 1B) and significant increase in diastolic ventricular diameter (Figure 1C), as signs of heart deterioration. Representative strain ultrasound images showing the movement of the left ventricle wall is showing (Figure 1D).

**Figure 2:**
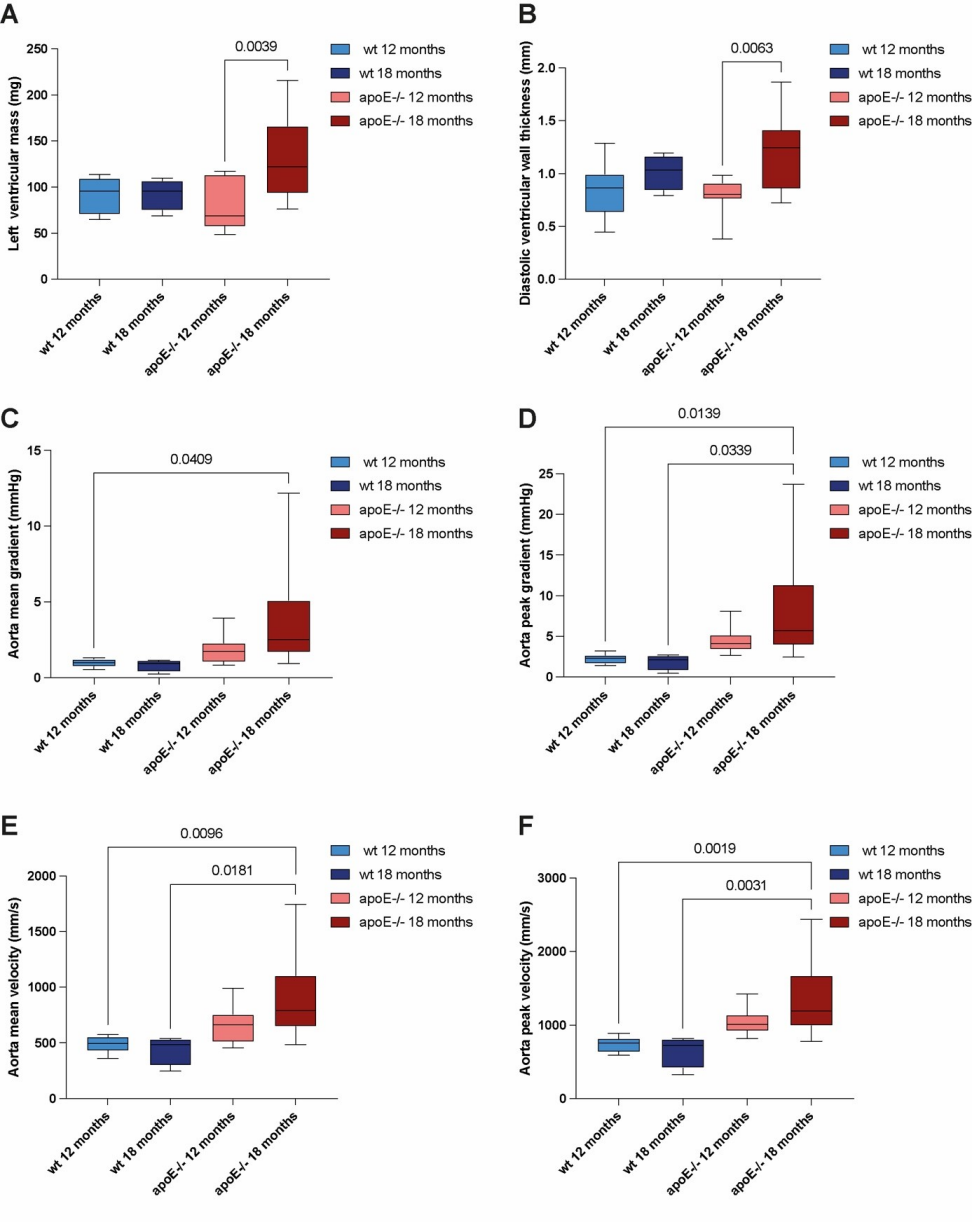
Hypertrophic changes in the heart of old apoE-/- mice (**A**) Left ventricular mass, (**B**) Diastolic ventricular wall thickness, (**C**) Aortic mean gradient, (**D**) Aortic peak gradient, (**E**) Aortic mean velocity and (**F**) Aortic peak velocity in mice measured at 12 and 18-months old. Significant *p *values are depicted in each figure.

**Figure 3:**
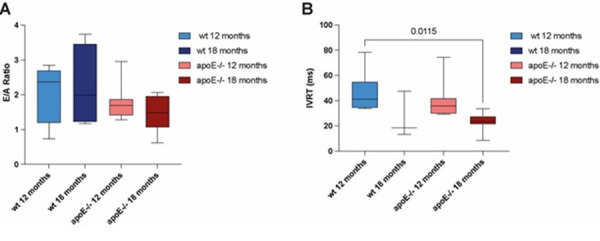
Assessment of diastolic disfunction (**A**) E/A ratio and (**B**) IVRT measured in mice at 12 and 18-months old. Significant p values are depicted in each figure.

Interestingly, only the old apoE^-/-^ mice developed ventricular hypertrophy during aging, demonstrated by increased left ventricular mass (Figure 2A) and left ventricular wall thickness (Figure 2B). Furthermore, the mean and peak gradients (Figure 2C,D) and the mean and peak velocities (Figure 2E,F) over aortic valve were significantly increased in old apoE^-/-^ mice. Since there were no signs of calcification or obstruction of valve leaflets, these changes can be explained by increased blood pressure over time in these mice. The E/A ratio was not significantly different between the groups and no clear sign of diastolic dysfunction was noted in these mice (Figure 3A). However, isovolumetric ventricular relaxation time (IVRT) was decreased in old apoE^-/-^ compare wildtype mice (Figure 3B), as a sign of heart failure with preserved heart function.

**Figure 4:**
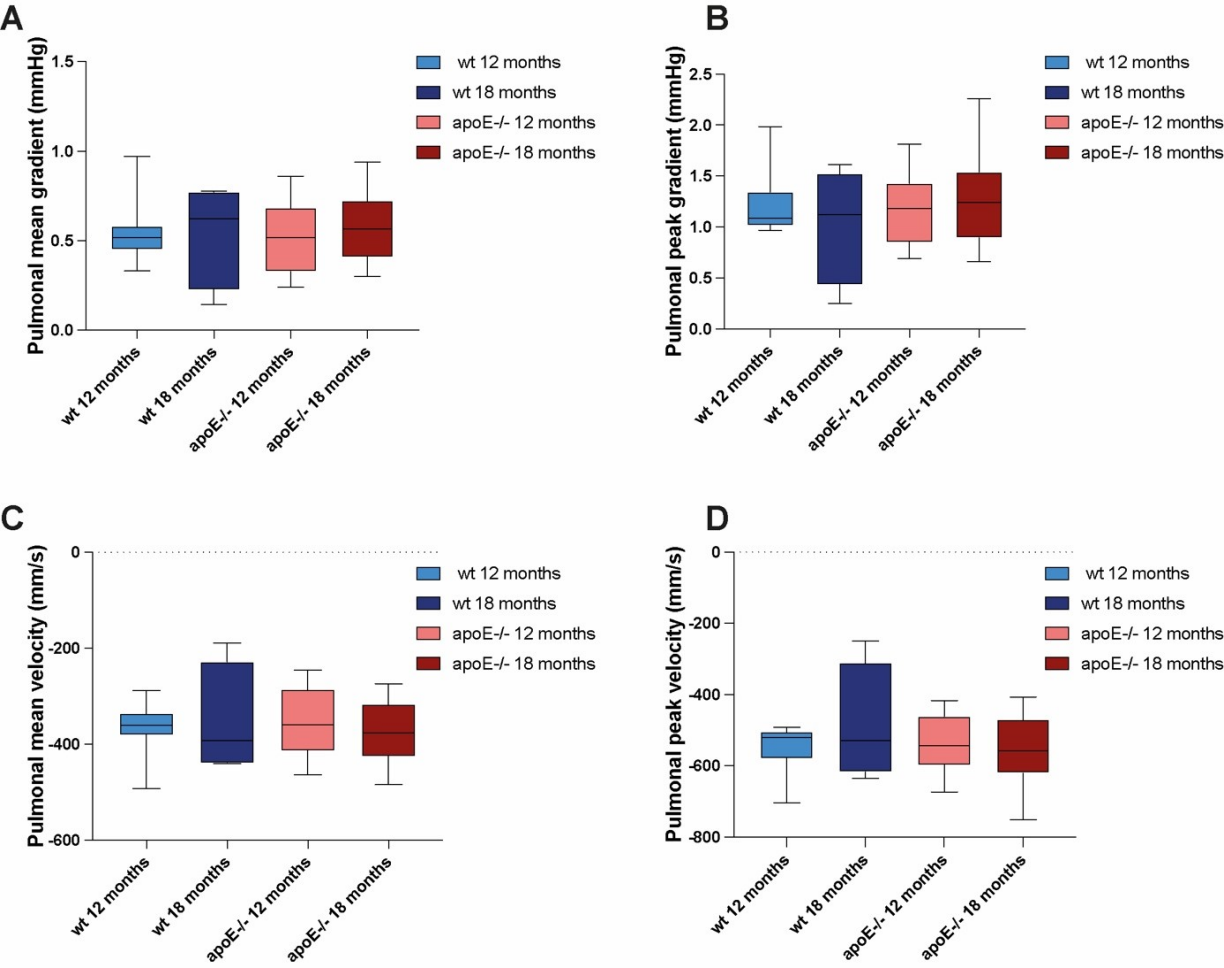
Assessment of pulmonary artery valve gradients (**A**) Pulmonal mean gradient, (**B**) Pulmonal peak gradient, (**C**) Pulmonal mean velocity and (**D**) Pulmonal peak velocity measured at 12 and 18-months old. Significant p values are depicted in the respective figures.

The gradients and velocity over pulmonary artery, as predicted by doppler echocardiographic pulmonary artery pressure, did not differ between the two groups or during aging (Figure 4), corresponding to a normal and unchanged right ventricular function.

Overall, our data showed that apoE deficient mice develop mild ventricular dysfunction during aging, even in the absence of high fat diet, which progresses towards heart failure with preserved EF.

## DISCUSSION

In this study, we have shown that deficiency of apoE could induce heart failure with preserved heart function by development of hypertrophic cardiomyopathy during ageing. Interestingly, we did not observe any changes in right ventricular function, despite the recent data in this regard^20^.

In the context of the cardiovascular diseases, the generation of genetically engineered mouse models became necessary to understand disease mechanisms in humans. Due to its role in lipid metabolism, apoE deficient mice (apoE-/-) became the main animal model to study atherosclerosis in cardiovascular research^21-24^. While the apoE knock-out mouse is an excellent model of atherosclerosis, the lack of apoE is extremely rare in the human population. As wild-type mouse apoE is equivalent to human apoE3 isoform, it is reasonable to speculate that human missing apoE3 (the presence of apoE2 and apoE4 isoforms), shares similar effects with ApoE deficient mice^2,6^.

Recently, many studies showed that the frequency of the ε4 allele is significantly increased in patients with dilated cardiomyopathy^16,20,25^, so the ε4 allele can be considered a novel risk factor for this non-ischemic cardiomyopathy. The possible mechanisms for inducing severe forms of dilated cardiomyopathy in ε4 allele carriers still need to be investigated. It can be speculated that repetitive inflammation^26^, apoptotic processes^8^, reduced antioxidant activity^9^, increased cell proliferation^7^ or excessive intracellular lipid accumulation^20,27-30^ induced by apoE4 modify structurally and functionally the heart, thus inducing cardiomyopathy and heart failure.

In the present study we have found changes in ventricular morphology, such as mass and thickness, which were associated with signs of diastolic dysfunction, but preserved ejection fraction. This is in accordance with recent studies which demonstrated that lipid accumulation into the heart induced increased catecholamines and angiotensin II release^31-33^, thus increasing the blood pressure and inducing ventricular hypertrophy. Since the ejection fraction was preserved, and no signs of diastolic disfunction were seen, we assumed that this could also be considered as a model of heart failure with preserved ejection fraction^34-36^. As a limitation of our study, we didn’t use high fat diet, which would have increased lipid accumulation and tissular damages and thus, aggravated the heart failure.

Further, in our recent study we have observed a different pattern in structural changes in right and left ventricles induced in apoE^-/-^ rats during aging and high fat diet^20^. Similar, humans carrying ε4 allele presented severe decrease in right ventricular function and increase in pulmonary arterial hypertension^20^. Other experimental studies described the development of atherosclerosis with severe pulmonary arterial hypertension in apoE^-/-^ mice feeding high fat diet^37-39^. In the present study we didn’t observe any changes in the right ventricular function, due probably to the still young age of the mice and normal diet during the experimental period.

In conclusion, apoE deficiency can induce ventricular dysfunction. However, in the absence of high fat diet, the mild changes are followed by development of heart failure with preserved ejection fraction. This is an interesting observation, since the cardiovascular scientific community is missing an established experimental animal model for this kind of pathology^40^. However, other studies are necessary to conclude over the role of apoE in cardiac physiology and its involvement in development of heart failure.

## KEY POINTS

**ApoE**

**◊** prevents diastolic dysfunction

**◊** prevents heart failure with preserved ejection fraction
